# Factors Affecting Dental Implant Failure: A Retrospective Analysis

**DOI:** 10.3390/healthcare13121356

**Published:** 2025-06-06

**Authors:** Raed AlRowis, Faris Albelaihi, Hamad Alquraini, Saud Almojel, Alwaleed Alsudais, Razan Alaqeely

**Affiliations:** 1 Department of Periodontics and Community Dentistry, College of Dentistry, King Saud University, Riyadh 11545, Saudi Arabia; 2 Dental Intern, College of Dentistry, King Saud University, Riyadh 11545, Saudi Arabia

**Keywords:** dental implants, osseointegration, implant failure

## Abstract

**Objectives:** This study aims to investigate the underlying causes of dental implant failure, focusing on implant-related complications and associated risk factors. Understanding these factors will help improve treatment planning and enhance implant success rates. **Methods:** A retrospective case-control study was conducted using clinical, medical, surgical, and radiographic records of patients who underwent dental implant removal due to complications. Key factors analyzed included patient-related variables (age, gender, medical conditions, periodontal disease), implant-related factors (implant site, implant system, restoration status), and procedural aspects (previous surgical interventions and reasons for implant removal). **Results:** The findings revealed that implant type (*p* = 0.004) and type of restoration (*p* = 0.001) significantly influenced implant survival. Gender (*p* = 0.001), medical conditions, smoking status (*p* = 0.004), and restoration status (*p* = 0.005) were significantly associated with specific failure mechanisms. Lack of osseointegration (36.4%) and absence of primary stability (22.4%) were the predominant causes of implant failure. Prior surgical interventions (*p* = 0.001) and decisions for re-implantation (*p* = 0.005) significantly affected implant removal frequency. **Conclusions:** Implant survival is influenced by multiple factors, with implant type, restoration type, and gender playing key roles in failure outcomes. Patient-specific risk assessment, particularly regarding medical conditions and smoking, meticulous surgical technique, and appropriate prosthetic planning, is vital for improving implant longevity and minimizing failure rates.

## 1. Introduction

In contemporary dentistry, dental implants are widely recognized as an effective and reliable method for replacing missing teeth in fully and partially edentulous patients. Dental implants provide functional and aesthetic benefits by making them a preferred option for oral rehabilitation, but various factors, including medication use, surgical procedures, and postoperative care, influence their success. According to Pjetursson et al., dental implants have a mean survival rate of 93.1% after a 10-year follow-up period [1]. High success rates have been associated with dental implants [2], which are not exempt from failures and complications affecting their long-term prognosis [3]. Generally, these failures are classified into biological and prosthetic complications [4,5].

Biologically, failures occur when the host tissue fails to achieve osseointegration with the dental implant, resulting in either early or late implant losses [6]. Early failures happen before or during the healing abutment stage. These are mainly due to a lack of adequate osseointegration [7]. In contrast, late failures involve the loss of an already integrated implant due to various causes, such as occlusal overloading or peri-implantitis [5,8]. On the other hand, prosthetic complications include mechanical issues of the implant components or the prosthetic structures, including screw loosening, chipping, and retention loss [9,10].

Various systemic and local factors contribute to implant failure, including systemic conditions such as diabetes [11], smoking [12], and medication usage, which can potentially negatively affect wound healing and osseointegration [13,14]. Whereas some studies have proven the adverse effects of these conditions on implant survival, others have produced conflicting findings about their implications [12]. Specifically, literature has proven inconsistent relationships between implant failure and conditions like controlled diabetes, moderate smoking, and the use of certain medications [15,16].

Recent literature reports associations between various medications and implant failure rates, including proton pump inhibitors (PPIs), nonsteroidal anti-inflammatory drugs (NSAIDs), selective serotonin reuptake inhibitors (SSRIs), glucocorticoids, and bisphosphonates [17]. The altered drug mechanisms of osseointegration include changing bone metabolism, disrupted wound healing, and inflammatory responses [18].

Local factors include the implant site, surgical technique, and prosthetic considerations [19]. Improper implant placement [20], poor bone quality [21], and previous surgical procedures in that area, like bone grafting or sinus lifts [22], are some of the causes that can increase the chances of complications [23].

Based on the 2017 World Workshop on the Classification of Periodontal and Peri-implant Diseases and Conditions consensus report, there is robust evidence that patients with a history of severe periodontitis, poor plaque control, and absence of regular maintenance care following implant therapy are at higher risk of developing peri-implantitis [24]. Yet, evidence pointing to smoking and diabetes as possible risk indicators for peri-implantitis is still inconclusive [24].

Although current dental implants have a favorable long-term prognosis, complications may necessitate their removal. However, long-term follow-up often does not occur at the same facility where the implant was placed, making it difficult to determine the true incidence and causes of implant failures [25]. Furthermore, little is known about patient outcomes following implant removal and whether re-implantation or alternative prosthetic rehabilitation is pursued.

This study aims to investigate the reasons for dental implant removal and the impact of various systemic, surgical, and prosthetic factors on implant survival. It also aims to identify the patient-specific biological factors and procedural variables that are the predominant determinants of multiple implant failures in the same individual. By analyzing clinical records and identifying key risk factors, this research seeks to provide valuable insights that can help clinicians improve treatment planning, reduce failure rates, and optimize long-term patient outcomes.

***Hypotheses.*** H_0_: Implant complications and associated baseline factors do not significantly influence the likelihood of implant removal, time required for implant failure, or overall implant survival. H_1_: Implants with complications are more likely to be lost. H_2_: At least one baseline factor, including year group, gender, medical conditions, periodontal disease, implant area, previous procedures, implant width, length, restoration status, reasons for removal, prosthetic use after removal, and re-implantation, significantly affects the implant failure. H_3_: There is a significant impact of various systemic, surgical, and prosthetic factors on implant survival. H_4_: Patient-specific biological factors, rather than procedural variables, are the significant predominant determinants of multiple implant failures in the same individual.

## 2. Materials and Methods

***Study Design.*** A retrospective case-control study was designed by evaluating patient records to identify factors contributing to dental implant failure, following the STROBE (Strengthening the Reporting of Observational Studies in Epidemiology) guidelines for observational research [26]. The study was conducted according to the Declaration of Helsinki and received ethical approval from the IRB at King Saud University Medical City (Project No E-21-5835 on 9 October 2024).

***Study Population.*** Data was retrieved for the patients from the Graduate Dental Clinic at University Dental Hospital, including all those who had undergone dental implant removal due to failure between 2017 and 2022. The population studied was purposely composed of subjects with varying age groups and health backgrounds to give a wide-based analysis.

***Sample Size.*** The sample size was calculated using G*Power software version 3.1.9.7, where the alpha level of significance is 0.05, the effect size is 0.15 (medium), and the power is 0.9 [27]. With these parameters, a total sample size of at least 130 failed implants was estimated to have good statistical power to carry out the hypothesis testing for this study.

***Inclusion and Exclusion Criteria.*** Patients were considered if at least one dental implant had been removed due to failure between 2017 and 2022, and the medical and radiographic records were complete for evaluation. In addition, pre-removal radiographs or CT images were required for assessment. Exclusion criteria included incomplete medical records and unclear or distorted radiographs, so adequate assessment could not be performed.

***Variables Analyzed.*** The study utilized various variables to examine their association with implant failure. Patient-related factors included age, gender, systemic diseases, periodontal disease, and smoking habits. Implant-specific factors encompass the implant site, implant length and width, and the restoration status. Surgical and prosthetic factors, such as prior surgical procedures, including bone grafting, sinus lift, and immediate placement, were also analyzed. Furthermore, the absence of maintenance appointments following implant therapy was noted as a possible risk factor for peri-implantitis. The main reasons for implant failure, which included infection, lack of osseointegration, improper implant positioning, trauma, and undetermined causes, were recorded and assessed.

***Data Collection and Statistical Analysis.*** Patient data were extracted from medical, surgical, and radiographic records, ensuring accurate categorization and coding for statistical analysis. Three calibrated operators were involved in data collection with an inter-examiner agreement of >90%. Each record was meticulously reviewed to maintain consistency and reliability. Descriptive statistics were used to summarize baseline characteristics and frequency distributions. Shapiro–Wilk tests were performed to assess the normality of continuous variables. Cox regression survival analysis was applied to estimate implant survival probabilities and evaluate differences between groups; chi-square tests examined associations between categorical variables and implant failure rates in the same individual multiple times. Missing data were handled through pairwise deletion for specific analyses, which explains the variation in sample sizes across different variables. The alpha significance level was set at 0.05, and any statistical test with a *p*-value less than 0.05 was considered statistically significant [28]. Data analysis was performed using IBM SPSS 27.

## 3. Results

***Baseline summary.*** In Table 1, the dataset comprises 132 implant cases. The distribution of cases over the years shows a relatively even spread. Gender representation is nearly balanced, with 52.3% males and 47.7% females, and the mean age is 58 ± 11 years (32–78 years).

Regarding health status, 59.8% of patients had no medical condition or smoking history, while 34.1% had a medical condition, and 6.1% were smokers. The most common medical conditions included hypertension (15.2%), controlled diabetes mellitus (9.8%), hypothyroidism (4.5%), and osteoporosis (3.0%). The most frequently used medications were antihypertensives (13.6%), oral hypoglycemics (8.3%), levothyroxine (4.5%), PPIs (3.8%), and bisphosphonates (2.3%) (Table 2).

Among periodontal conditions, 47.0% of patients had no periodontal disease, 44.7% had periodontal disease, and 8.3% were fully edentulous. The total number of implants varied, with 26.5% of cases having 0–1 implants, 47.7% having fewer than five, and 25.8% having more than five implants. The implant locations were diverse, with the lower molar area being the most common site (27.3%), followed by the upper molar area (18.9%) and the upper premolar area (18.2%).

Regarding prior surgical interventions, 60.3% of cases had no prior surgical intervention at the implant site, while 30.2% had a bone graft, and 4.8% each had sinus lifting or were immediate implantation. Implant length was most frequently 10 mm (63.4%), with 23.7% having implants shorter than 10 mm and 13.0% longer than 10 mm. Implant width was primarily regular (46.6%), followed by narrow (44.3%) and wide (9.2%). The most common implant type was regular bone level (78.7%), with a smaller proportion being regular tissue level (18.1%) and tapered bone level (3.1%).

Most implants (81.1%) failed before restoration, while 19.0% were successfully restored. Among those that were restored, the most common types of restorations included crowns (10.6%), fixed dental prostheses (FDPs) (6.06%), and overdentures (ODs) (2.3%). Regarding the reasons for implant removal, the most common were lack of osseointegration (36.4%) and absence of primary stability (22.4%), followed by peri-implantitis (14.0%), iatrogenic issues related to location or position (14.0%), and infection (7.5%). Less frequent reasons included non-functionality (implants that achieved osseointegration but could not be prosthetically loaded due to inappropriate positioning) (3.7%) and prosthetic trauma (1.9%).

After implant removal, 63.6% of patients chose to have another implant, while 35.7% decided against it. Surgeon experience included periodontal consultants (37.1%), oral and maxillofacial surgery (OMFS) consultants (25.0%), periodontal postgraduates (28.0%), and OMFS postgraduates (9.8%). Only 36.4% of patients attended regular maintenance appointments, leaving 63.6% without consistent follow-up care. This data offers a comprehensive overview of patient characteristics, implant information, removal reasons, and follow-up decisions, enabling a deeper examination of factors that influence implant success and failure.

***Association between baseline summary and dental implant failure.*** Table 3 presents a detailed investigation of reasons for dental implant removal across various patient and implant-related factors. The analysis identified three statistically significant associations among the variables studied: gender (*p* = 0.001), medical conditions, and smoking (*p* = 0.004). Gender differences were pronounced in our sample of 107 implants. Of the 58 implants in men, failures were primarily due to no osseointegration (20.6% of total implants), followed equally by peri-implantitis and no primary stability (11.2% each). Among the 49 implants in women, failures were most commonly due to no osseointegration (15.9%), followed by iatrogenic causes (10.3%) and no primary stability (11.2%). The stark difference in peri-implantitis (11.2% in men vs. 2.8% in women) and iatrogenic failures (3.7% in men vs. 10.3% in women) contributed to the statistical significance. Medical conditions and smoking status strongly influenced failure patterns. In the 66 implants without medical conditions, failures were primarily due to no osseointegration (19.6%) and no primary stability (17.8%). Among the 35 implants with medical conditions, no osseointegration was the most common (13.1%), followed by peri-implantitis (6.5%). Despite representing only six implants, smokers showed remarkably high rates of osseointegration failure (3.7% of total implants) and infection (1.9%). Also, in the 21 restored implants, most failed from peri-implantitis (5.6%) and iatrogenic causes (5.6%), with relatively low rates of osseointegration failure (1.9%). Although not statistically significant, notable trends included higher peri-implantitis rates in the 46 patients with periodontal disease (9.3%) compared to the 54 patients without periodontal disease (2.8%). Implant location showed variations, with upper anterior implants having higher rates of peri-implantitis (2.8% of total). Of the 40 implants placed by periodontist consultants, a high rate of osseointegration failure was observed (18.7% of total implants). This comprehensive analysis highlights the multifactorial nature of implant failure and identifies key risk factors that clinicians should consider when planning implant treatments.

***Cox regression analysis of factors affecting dental implant survival.*** This test was applied to check the impact of various systemic, surgical, and prosthetic factors on the implant. Table 4 presents a comprehensive Cox regression analysis examining the effect of various systemic, surgical, and prosthetic factors on dental implant survival. The analysis revealed that among all factors studied, only statistically significant predictors were implant type (*p* = 0.004) and type of restoration (*p* = 0.001). Regular tissue-level implants showed a trend toward better survival than tapered bone level implants (Exp(B) = 0.373, *p* = 0.055). For restoration types, overdentures (OD) showed a 3.44 times higher risk of failure (*p* = 0.027), and crowns showed a 2.42 times higher risk (*p* < 0.001). Surprisingly, implant dimensions (length and width) and surgeon experience did not significantly affect implant survival. The overall model was statistically significant (Chi-square = 57.048, *p* = 0.001), demonstrating that these collective factors effectively predict implant survival, with surgical and prosthetic factors having greater influence than systemic or dimensional factors.

***Determinants of multiple implant failures in the same individual.*** The results presented in Table 5 highlight significant and marginally significant associations between various factors and the number of times dental implants were removed. A strong association is observed between prior surgical intervention and implant removal times, as indicated by a highly significant chi-square value (χ^2^ = 34.797, *p* = 0.001). A marginally significant relationship is found between medical conditions combined with smoking status and implant removal times (χ^2^ = 14.431, *p* = 0.071), implying a potential influence of these factors, though not reaching conventional significance thresholds. Similarly, the area of implant placement shows a marginal association (χ^2^ = 28.987, *p* = 0.088), indicating that the location of implantation might play a role in removal frequency, albeit with weaker statistical support. These findings suggest that prior interventionχ in the implantation area and the decision to undergo implantation again are the most influential factors in implant removal frequency. In contrast, medical conditions, smoking, and implant location show weaker but still noteworthy associations.

## 4. Discussion

This retrospective analysis of dental implant failures reveals essential insights into factors influencing implant survival and complications. Our findings demonstrate that implant type and type of restoration significantly impact implant survival outcomes. These results align with previous studies, which identified similar critical factors in implant success rates [3,4].

The anatomical location of implant placement emerged as a potential determinant of survival with a marginally significant association. The lower molar area was the most common implantation site (27.3%), followed by the upper molar (18.9%) and the upper premolar (18.2%). This finding corresponds with Pjetursson et al.’s finding that reported variable success rates across different oral regions [1]. The favorable outcomes in the anterior maxilla were attributed to better bone quality and quantity [29], though our sample size for this region was relatively small (12.2% of cases). These location-specific differences highlight the importance of careful site selection and preparation, as Karlsson et al. emphasized in their technical complication analysis [9]. Like that, the relationship between implant location and failure risk is due to the biomechanical stress distribution and local bone quality. The marginally significant association between implant area and removal frequency is that implants placed in regions with lower bone density experience higher failure rates due to compromised primary stability [3]. While our finding that the lower molar area was the most common implantation site (27.3%) reflects common clinical practice, it also highlights the challenges of achieving optimal outcomes in posterior regions where masticatory forces are highest.

Our analysis revealed that lack of osseointegration and absence of primary stability were the predominant causes of implant failure, followed by peri-implantitis, iatrogenic issues, and infection, supporting observations by Esposito et al., regarding the primary biological factors in implant loss [5]. The significantly shorter survival times in cases with poor primary stability highlight the critical importance of initial mechanical engagement [14,30,31]. Notably, restoration status showed a significant association with reasons for removal, as they mostly failed due to peri-implantitis and prosthetic trauma because of the shift in failure etiology from biological (osseointegration) to mechanical and inflammatory (peri-implantitis, prosthetic trauma) following restoration. This transition from initial healing failures to load-related complications aligns with Gandzo et al. (2023) study, which found that the classification of implant complications distinguished between early biological complications and late technical/biological failures [32].

The influence of medical conditions and smoking on implant survival presents significant findings in our study. Patients with medical conditions experienced higher rates of peri-implantitis (26.8% vs. 8.8% in healthy patients), while smokers showed remarkably high rates of osseointegration failure and infection. This finding is consistent with previous studies that identified these factors as risk indicators [33,34] and reflects the impact of systemic conditions on wound healing and osseointegration [3,35] because systemic inflammatory conditions create a proinflammatory microenvironment that accelerates peri-implant bone loss. These biological mechanisms provide a logical foundation for the statistical associations observed in our study.

However, it is essential to acknowledge that the literature presents some contradictory findings regarding the impact of systemic conditions on implant survival. Several studies have shown less definitive associations between certain medical conditions and implant failure rates [16,36,37]. The 2017 World Workshop consensus on risk indicators for peri-implantitis concluded that while some associations exist, data identifying smoking and diabetes as potential risk indicators remain inconclusive [24]. These conflicting findings highlight the complex interplay between systemic conditions and local factors influencing implant outcomes.

Gender differences also significantly influenced failure patterns, with men experiencing considerably higher rates of peri-implantitis, while women had more iatrogenic failures and non-functional implants. The significant variation in outcomes based on surgical expertise, though not statistically significant in our analysis, aligns with previous research emphasizing the importance of surgical experience [38]. However, this finding should be interpreted within the context of potential case selection bias and varying complexity levels assigned to different practitioners [39].

The significant gender-based differences in failure patterns may reflect biological and behavioral factors. The higher prevalence of peri-implantitis in male patients aligns with Derks et al.’s (2016) large-scale study, which identified male gender as an independent risk factor for peri-implantitis, potentially due to differences in oral hygiene practices and inflammatory responses [40]. Conversely, the higher rate of iatrogenic failures in female patients may be related to anatomical factors such as typically thinner alveolar ridges and reduced bone density in female patients [41].

These gender-specific risk profiles underline the importance of personalized treatment planning based on comprehensive risk assessment. The impact of surgical expertise on implant success has been well-documented in the literature; however, standardized protocols will help to minimize operator-dependent variations [42,43] because repeated surgeries compromise vascularization and cellular activity in the surgical site, potentially limiting regenerative capacity [40]. Along with that, a strong association was observed between prior surgical intervention and implant removal times (χ^2^ = 34.797, *p* = 0.001), and the decision to implant again was significantly associated with implant removal times (χ^2^ = 28.252, *p* = 0.005). These findings suggest that prior surgical interventions and subsequent treatment decisions significantly impact implant outcomes. As Park et al. (2022) noted, the decision to re-implant following failure should be guided by a thorough analysis of the initial failure mechanism and the potential for site remediation [41]. The marginally significant relationship between medical conditions and smoking and removal frequency aligns with Mosaddad et al.’s (2024) conclusion that conditions create a persistent state of compromised healing that affects multiple interventions over time [44].

As to the effect of maintenance, only 36.4% of patients reported regular maintenance visits, whereas 63.6% failed to adhere to suggested follow-up treatment. While we did not explicitly examine the statistical correlation between compliance with maintenance and implant failure, this finding is consistent with the agreement of the 2017 World Workshop, which cited failure to receive regular maintenance care as a significant risk factor for peri-implantitis onset. This highlights the extreme significance of emphasizing post-implant care protocols for patients.

Several limitations must be acknowledged when interpreting these results. The retrospective design inherently introduces potential recall bias and documentation inconsistencies [45]. The focus on failed implants without a control group of successful cases and the single-center nature of the study limits our ability to establish definitive risk factors [46].

While these are limitations, our current outcomes add to the increasing body of evidence guiding implant therapy. In particular, the results have underlined the complex interaction between biological, prosthetic, and practitioner-related factors in determining implant success. The Cox regression analysis demonstrated that surgical and prosthetic factors have greater influence than systemic or dimensional factors, with removal technique and restoration type being particularly significant predictors of implant survival.

Looking ahead, these findings point to several avenues for future investigation. Prospective, multi-center studies with matched control groups will go a long way in determining more definitive causal relationships between risk factors and implant failure. Exploring local anatomical factors in greater depth and standardized reporting of medical conditions will further elucidate the failure mechanisms. Moreover, further investigation of the impact of gender differences and previous surgical interventions will provide helpful insight into risk management strategies.

Concerning our initial hypotheses, we concluded that (1) our hypothesis regarding the impact of systemic conditions on implant failure was partially confirmed, with medical conditions showing significant associations with failure patterns (*p* = 0.004), particularly peri-implantitis. (2) The hypothesis that anatomical location influences implant survival was supported by the marginally significant association (*p* = 0.088) between implant area and removal patterns. (3) The hypothesis concerning surgical expertise was not statistically confirmed (*p* = 0.452), though trends in the data suggested some influence of operator experience. (4) The hypothesis that restoration type affects implant survival was strongly confirmed (*p* = 0.001), with overdentures and crowns showing significantly higher failure risks. (5) Our findings indirectly supported the hypothesis regarding maintenance that 63.6% of patients with failed implants did not attend regular maintenance appointments.

## 5. Conclusions

Our retrospective examination of dental implant failure revealed several significant factors linked to implant survival and failure mechanisms. Implant type and restoration type were the statistically significant predictors of implant survival, whereas gender and medical conditions had a significant effect on failure mechanisms. A marginally substantial effect was seen on implant outcomes if anatomical location was used. The most common failure causes—the absence of osseointegration and lack of primary stability—emphasize the paramount significance of meticulous treatment planning and accurate surgical technique.

Although our study sheds essential light on factors impacting implant survival, the results still need to be interpreted within the context of clinical practice realities and patient-specific factors. The findings underline the need for cautious patient selection, meticulous surgical technique, and appropriate timing of prosthetic rehabilitation. Future research addressing the identified limitations would further enhance our understanding of the mechanisms of implant failure and help in treatment outcome optimization across a diverse range of patient populations.

## Figures and Tables

**Table 1:** Baseline summary of demographic and clinical characteristics.

Characteristic	*f*	*%*
**Age groups**		
Less than 40	33	24.5
40–59	63	47.6
60 and more	36	26.9
**Gender**		
Male	69	52.3
Female	63	47.7
**Medical Condition + Smoking**		
Absent	79	59.8
Present	45	34.1
Smokers	8	6.1
**Periodontal Disease**		
No	62	47.0
Yes	59	44.7
Edentulous	11	8.3
**Total Implants**		
0–1	35	26.5
Less than 5	63	47.7
More than 5	34	25.8
**Area of Implant**		
Upper Molar	25	18.9
Upper Premolar	24	18.2
Upper Anterior	17	12.9
Lower Molar	36	27.3
Lower Premolar	18	13.6
Lower Anterior	12	9.1
**Prior surgical intervention**		
None	76	60.3
Bone Grafting	38	30.2
Sinus Lifting	6	4.8
Immediate Implant	6	4.8
**Implant Length**		
10 mm	83	63.4
More than 10 mm	17	13.0
Less than 10 mm	31	23.7
**Implant Width**		
Regular	61	46.6
Narrow	58	44.3
Wide	12	9.2
**Type of Implant**		
Regular Bone Level	100	78.7
Regular Tissue Level	23	18.1
Tapered Bone Level	4	3.1
**Was implant restored?**		
No	107	81.1
Yes	25	19.0
**Type of restoration for restored implants**		
Crown	14	10.6
FDP	8	6.06
OD	3	2.3
**Reason for implant removal**		
No Osseointegration	39	36.4
No Primary Stability	24	22.4
Peri-implantitis	15	14.0
Iatrogenic or non-functional	19	17.7
Infection	8	7.5
Prosthetic Trauma	2	1.9
**Implant Again after failure**		
Yes	82	63.6
No	46	35.7
Yes, but in different location	1	0.8
**Surgeons Experience**		
Perio Consultant	49	37.1
OMFS Consultant	33	25.0
Perio PG	37	28.0
OMFS PG	13	9.8
**Regular Maintenance**		
Yes	48	36.4
No	84	63.6

Note. Total N = 132. FDP = fixed dental prosthesis; OD = overdenture; OMFS = oral and maxillofacial surgery; PG = postgraduate, f= frequency.

**Table 2:** Specific medical conditions and medications of patients.

Medical Conditions	n	%	Medications	n	%
Hypertension	20	15.2	Antihypertensives	18	13.6
Controlled Diabetes Mellitus	13	9.8	Oral Hypoglycemics	11	8.3
Hypothyroidism	6	4.5	Levothyroxine	6	4.5
Osteoporosis	4	3.0	PPIs	5	3.8
Rheumatoid Arthritis	3	2.3	Bisphosphonates	3	2.3
Cardiovascular Disease	3	2.3	NSAIDs (chronic use)	3	2.3
Asthma	2	1.5	Corticosteroids	2	1.5
Depression	2	1.5	SSRIs	2	1.5
Other	3	2.3	Other	6	4.5

Note: Some patients had multiple conditions and medications.

**Table 3:** Reasons for dental implant removal across all patient and implant-related factors.

Reason for Removal	No Primary Stability	No Osseointegration	Peri-Implantitis	Infection	Not Functional	Iatrogenic	Prosthetic Trauma	*χ^2^*	*df*	*p*
**Age**								11.73	12	0.46
Less than 40	8 (28.6)	9 (32.14)	3 (10.71)	3 (10.71)	0 (0.0)	5(17.8)	0 (0.0)			
40–59	12 (23.08)	16 (30.77)	11 (21.15)	4 (7.69)	3 (5.77)	4 (7.69)	2 (3.85)			
60 and more	5 (16.67)	6 (20.0)	5 (16.67)	3 (10.0)	1 (3.33)	7 (23.33)	3 (10.0)			
**Gender**								22.850	6	0.001 *
Male	12 (11,2)	22 (20.6)	12 (11.2)	6 (5.6)	1 (0.9)	4 (3.7)	1 (0.9)			
Female	12 (11.2)	17 (15.9)	3 (2.8)	2 (1.9)	3 (2.8)	11 (10.3)	1 (0.9)			
**Medical Condition + Smoking**								29.311	12	0.004 *
Absent	19 (17.8)	21 (19.6)	8 (7.5)	4 (3.7)	4 (3.7)	9 (8.4)	1 (0.9)			
present	5 (4.7)	14 (13.1)	7 (6.5)	2 (1.9)	0 (0.0)	6 (5.6)	1 (0.9)			
Smoking	0 (0.0)	4 (3.7)	0 (0.0)	2 (1.9)	0 (0.0)	0 (0.0)	0 (0.0)			
**Periodontal Disease**								17.946	12	0.117
No	18 (16.8)	20 (18.7)	3 (2.8)	3 (2.8)	2 (1.9)	6 (5.6)	2 (1.9)			
Yes	5 (4.7)	17 (15.9)	10 (9.3)	5 (4.7)	1 (0.9)	8 (7.5)	0 (0.0)			
Edentulous	1 (0.9)	2 (1.9)	2 (1.9)	0 (0.0)	1 (0.9)	1 (0.9)	0 (0.0)			
**Area of Implant**								43.723	30	0.051
Upper Molar	6 (5.6)	10 (9.3)	1 (0.9)	0 (0.0)	0 (0.0)	2 (1.9)	1 (0.9)			
Upper Premolar	7 (6.5)	5 (4.7)	4 (3.7)	1 (0.9)	0 (0.0)	1 (0.9)	0 (0.0)			
Upper Anterior	1 (0.9)	6 (5.6)	3 (2.8)	2 (1.9)	1 (0.9)	2 (1.9)	0 (0.0)			
Lower Molar	7 (6.5)	6 (5.6)	3 (2.8)	4 (3.7)	2 (1.9)	8 (7.5)	1 (0.9)			
Lower Premolar	3 (2.8)	9 (8.4)	3 (2.8)	0 (0.0)	1 (0.9)	1 (0.9)	0 (0.0)			
Lower Anterior	0 (0.0)	3 (2.8)	1 (0.9)	1 (0.9)	0 (0.0)	1 (0.9)	0 (0.0)			
**Implant Length**								11.607	12	0.478
10 mm	13 (12.1)	24 (22.4)	11 (10.3)	4 (3.7)	1 (0.9)	12 (11.2)	1 (0.9)			
>10 mm	5 (4.7)	6 (5.6)	1 (0.9)	2 (1.9)	1 (0.9)	0 (0.0)	0 (0.0)			
<10 mm	6 (5.6)	9 (8.4)	3 (2.8)	2 (1.9)	2 (1.9)	3 (2.8)	1 (0.9)			
**Implant Width**								20.412	12	0.060
Regular	15 (14.0)	19 (17.8)	6 (5.6)	4 (3.7)	1 (0.9)	7 (6.5)	0 (0.0)			
Narrow	6 (5.6)	17 (15.9)	9 (8.4)	4 (3.7)	2 (1.9)	6 (5.6)	0 (0.0)			
Wide	3 (2.8)	3 (2.8)	0 (0.0)	0 (0.0)	1 (0.9)	2 (1.9)	2 (1.9)			
**Prior surgical intervention**								13.992	18	0.730
None	12 (11.4)	22 (21.0)	10 (9.5)	5 (4.8)	2 (1.9)	9 (8.6)	1 (1.0)			
Bone Graft	8 (7.6)	11 (10.5)	4 (3.8)	2 (1.9)	2 (1.9)	6 (5.7)	0 (0.0)			
Sinus Lifting	1 (1.0)	4 (3.8)	1 (1.0)	0 (0.0)	0 (0.0)	0 (0.0)	0 (0.0)			
Immediate Implant	3 (2.9)	1 (1.0)	0 (0.0)	1 (1.0)	0 (0.0)	0 (0.0)	0 (0.0)			
**Restoration Status**								18.513	6	0.005 *
Not Restored	19 (17.8)	37 (34.6)	9 (8.4)	7 (6.5)	3 (2.8)	9 (8.4)	2 (1.9)			
Restored	0 (0.0)	7 (6.6)	6 (5.6)	1 (0.9)	1 (0.9)	6 (5.6)	0 (0.0)			
**Implant Type**								14.037	12	0.298
Regular Bone Level	19 (18.3)	26 (25.0)	14 (13.5)	5 (4.8)	2 (1.9)	13 (12.5)	1 (1.0)			
Regular Tissue Level	4 (3.8)	9 (8.7)	0 (0.0)	2 (1.9)	2 (1.9)	2 (1.9)	1 (1.0)			
Tapered Bone Level	1 (1.0)	2 (1.9)	0 (0.0)	1 (1.0)	0 (0.0)	0 (0.0)	0 (0.0)			
**Surgeon’s Experience**								27.163	18	0.076
Periodontist Consultant	9 (8.4)	20 (18.7)	2 (1.9)	3 (2.8)	1 (0.9)	4 (3.7)	1 (0.9)			
OMFS Consultant	4 (3.7)	6 (5.6)	5 (4.7)	2 (1.9)	1 (0.9)	7 (6.5)	0 (0.0)			
Periodontist Postgraduate	7 (6.5)	10 (9.3)	5 (4.7)	3 (2.8)	1 (0.9)	2 (1.9)	1 (0.9)			
OMFS Postgraduate	4 (3.7)	3 (2.8)	3 (2.8)	0 (0.0)	1 (0.9)	2 (1.9)	0 (0.0)			

Note. OMFS = oral and maxillofacial surgery. * *p* < 0.05.

**Table 4:** Cox regression results for factors associated with dental implant survival.

Variable	B	SE	Wald	*df*	Sig.	Exp(B)
**Implant Length**			1.475	2	0.478	
10 mm	−0.117	0.229	0.260	1	0.610	0.890
More than 10 mm	−0.382	0.319	1.435	1	0.231	0.682
Less than 10 mm (ref.)	—	—	—	—	—	—
**Implant Width**			1.274	2	0.529	
Regular	0.391	0.361	1.175	1	0.278	1.478
Narrow	0.269	0.353	0.579	1	0.447	1.308
Wide (ref.)	—	—	—	—	—	—
**Type of Implant**			10.951	2	0.004 **	
Regular Bone Level	−0.131	0.473	0.077	1	0.782	0.877
Regular Tissue Level	−0.987	0.515	3.675	1	0.055	0.373
Tapered Bone Level (ref.)	—	—	—	—	—	—
**Type of Restoration**			18.057	4	0.001 **	
Crown	0.883	0.239	13.678	1	<0.001 ***	2.419
FDP	0.116	0.286	0.164	1	0.686	1.123
OD	1.236	0.558	4.902	1	0.027 *	3.442
Removable Denture	0.233	0.324	0.519	1	0.471	1.263
**Surgeons Experience**			2.630	3	0.452	
Perio Cons	0.287	0.331	0.753	1	0.386	1.333
OMFS Cons	0.434	0.351	1.524	1	0.217	1.543
Perio PG	0.496	0.334	2.203	1	0.138	1.642
OMFS PG (ref.)	—	—	—	—	—	—

Note. N = 164. -2 Log likelihood = 1326.036; chi-square = 57.048, *df* = 27, *p* = 0.001. The dependent variable is time difference, with implant status (1) as an event. Reference categories are indicated for categorical variables (ref.). * *p* < 0.05, ** *p* < 0.01, and *** *p* < 0.001.

**Table 5:** Significant associations between variables and dental implant removal times.

Variable	*χ^2^*	*df*	*p*	Interpretation
Prior Surgical Intervention	34.797	12	0.001 *	A significant association between previous work in the area and implant removal times
Implant Again	28.252	12	0.005 *	A significant association between the decision to implant again and implant removal times
Medical Condition + Smoking	14.431	8	0.071	A marginally significant association between medical condition/smoking status and implant removal times
Area of Implant	28.987	20	0.088	A marginally significant association between implant location and implant removal times

Note. Implant times refer to the number of times implants were placed (Time 1 to Time 5). Only variables with *p* < 0.10 are included in this table. * indicates statistical significance at *p* < 0.05.

## Data Availability

Data are available upon request from the corresponding author.

## References

[1] Pjetursson B.E., Thoma D., Jung R., Zwahlen M., Zembic A. (2012). A systematic review of the survival and complication rates of implant-supported fixed dental prostheses (fdps) after a mean observation period of at least 5 years. Clin. Oral Implant. Res..

[2] Oh S.-L., Shiau H.J., Reynolds M.A. (2020). Survival of dental implants at sites after implant failure: A systematic review. J. Prosthet. Dent..

[3] Chrcanovic B.R., Albrektsson T., Wennerberg A. (2014). Reasons for failures of oral implants. J. Oral Rehabil..

[4] Berglundh T., Persson L., Klinge B. (2002). A systematic review of the incidence of biological and technical complications in implant dentistry reported in prospective longitudinal studies of at least 5 years. J. Clin. Periodontol..

[5] Esposito M., Hirsch J., Lekholm U., Thomsen P. (1998). Biological factors contributing. Eur. J. Oral Sci..

[6] Insua A., Monje A., Wang H.-L., Miron R.J. (2017). Basis of bone metabolism around dental implants during osseointegration and peri-implant bone loss. J. Biomed. Mater. Res. Part A.

[7] Vinhas A.S., Aroso C., Salazar F., López-Jarana P., Ríos-Santos J.V., Herrero-Climent M. (2020). Review of the mechanical behavior of different implant–abutment connections. Int. J. Environ. Res. Public Health.

[8] Ishak M.I., Daud R., Ibrahim I., Mat F., Mansor N.N. (2022). Biomechanical overloading factors influencing the failure of dental implants: A review. Structural Integrity Cases in Mechanical and Civil Engineering.

[9] Karlsson K., Derks J., Håkansson J., Wennström J.L., Molin Thorén M., Petzold M., Berglundh T. (2018). Technical complications following implant-supported restorative therapy performed in Sweden. Clin. Oral Implant. Res..

[10] Sailer I., Karasan D., Todorovic A., Ligoutsikou M., Pjetursson B.E. (2022). Prosthetic failures in dental implant therapy. Periodontology 2000.

[11] Monje A., Kan J.Y., Borgnakke W. (2023). Impact of local predisposing/precipitating factors and systemic drivers on peri-implant diseases. Clin. Implant. Dent. Relat. Res..

[12] Rose L.F., Mealey B.L. (2015). Implant complications associated with systemic disorders and medications. Dental Implant Complications.

[13] Dutta S.R., Passi D., Singh P., Atri M., Mohan S., Sharma A. (2020). Risks and complications associated with dental implant failure: Critical update. Natl. J. Maxillofac. Surg..

[14] Alsaadi G., Quirynen M., Komárek A., Van Steenberghe D. (2007). Impact of local and systemic factors on the incidence of oral implant failures, up to abutment connection. J. Clin. Periodontol..

[15] Monje A., Catena A., Borgnakke W.S. (2017). Association between diabetes mellitus/hyperglycaemia and peri-implant diseases: Systematic review and meta-analysis. J. Clin. Periodontol..

[16] Marchio V., Derchi G., Cinquini C., Miceli M., Gabriele M., Alfonsi F., Barone A. (2020). Tissue level implants in healthy versus medically compromised patients: A cohort comparative study. Minerva Stomatol..

[17] Corbella S., Morandi P., Alberti A., Morandi B., Francetti L. (2022). The effect of the use of proton pump inhibitors, serotonin uptake inhibitors, antihypertensive, and anti-inflammatory drugs on clinical outcomes of functional dental implants: A retrospective study. Clin. Oral Implant. Res..

[18] Apostu D., Lucaciu O., Lucaciu G.D.O., Crisan B., Crisan L., Baciut M., Onisor F., Baciut G., Câmpian R.S., Bran S. (2017). Systemic drugs that influence titanium implant osseointegration. Drug Metab. Rev..

[19] Toffler M., Rosen P.S. (2015). Complications with transcrestal sinus floor elevation: Etiology, prevention, and treatment. Dental Implant Complications.

[20] Moy P.K., Aghaloo T. (2019). Risk factors in bone augmentation procedures. Periodontology 2000.

[21] Taban M., Fatemi A., Soleimani M., Sajedi S.M., Sabzevari B. (2023). Risk factors associated with implant sites prepared by orthodontic treatment: A systematic review. Eur. J. Transl. Myol..

[22] Hussain R.A., Gangwani P., Miloro M. (2022). Implant surgery. Management of Complications in Oral and Maxillofacial Surgery.

[23] Omar S., Jaiswal H., Kumar P., Mishra S.K. (2022). Surgical considerations and related complications in oral implantology: A comprehensive review. J. Prim. Care Dent. Oral Health.

[24] Berglundh T., Armitage G., Araujo M.G., Avila-Ortiz G., Blanco J., Camargo P.M., Chen S., Cochran D., Derks J., Figuero E. (2018). Peri-implant diseases and conditions: Consensus report of workgroup 4 of the 2017 World Workshop on the Classification of Periodontal and Peri-Implant Diseases and Conditions. J. Periodontol..

[25] Roy M., Loutan L., Garavaglia G., Hashim D. (2020). Removal of osseointegrated dental implants: A systematic review of explantation techniques. Clin. Oral Investig..

[26] Cevallos M., Egger M. (2014). Strobe (Strengthening the reporting of observational studies in epidemiology). Guidelines for Reporting Health Research: A User’s Manual.

[27] Kang H., Huh S. (2021). Sample size determination and power analysis using the G*Power software. J. Educ. Eval. Health Prof..

[28] Landau S., Everitt B.S. (2003). A Handbook of Statistical Analyses Using SPSS.

[29] Zhou Y., Gao J., Sheng M., Qi W., Jin J., He F. (2020). Facial alveolar bone alterations and gray value changes based on cone beam computed tomography around maxillary anterior implants: A clinical retrospective study of 1–3 years. Clin. Oral Implant. Res..

[30] Javed F., Ahmed H.B., Crespi R., Romanos G.E. (2013). Role of primary stability for successful osseointegration of dental implants: Factors of influence and evaluation. Interv. Med. Appl. Sci. IMAS.

[31] Huang Y.-C., Huang Y.-C., Ding S.-J. (2023). Primary stability of implant placement and loading related to dental implant materials and designs: A literature review. J. Dent. Sci..

[32] Gadzo N., Ioannidis A., Naenni N., Hüsler J., Jung R.E., Thoma D.S. (2023). Survival and complication rates of two dental implant systems supporting fixed restorations: 10-year data of a randomized controlled clinical study. Clin. Oral Investig..

[33] Chatzopoulos G.S., Wolff L.F. (2023). Dental implant failure and factors associated with treatment outcome: A retrospective study. J. Stomatol. Oral Maxillofac. Surg..

[34] Takamoli J., Pascual A., Martinez-Amargant J., Garcia-Mur B., Nart J., Valles C. (2021). Implant failure and associated risk indicators: A retrospective study. Clin. Oral Implant. Res..

[35] Bielemann A.M., Marcello-Machado R.M., Del Bel Cury A.A., Faot F. (2018). Systematic review of wound healing biomarkers in peri-implant crevicular fluid during osseointegration. Arch. Oral Biol..

[36] Schimmel M., Srinivasan M., McKenna G., Müller F. (2018). Effect of advanced age and/or systemic medical conditions on dental implant survival: A systematic review and meta-analysis. Clin. Oral Implant. Res..

[37] Carr A.B., Revuru V.S., Lohse C.M. (2017). Association of systemic conditions with dental implant failures in 6384 patients during a 31-year follow-up period. Int. J. Oral Maxillofac. Implant..

[38] Goldstein M., Donos N., Teughels W., Gkranias N., Temmerman A., Derks J., Kuru B.E., Carra M.C., Castro A.B., Dereka X. (2024). Structure, governance and delivery of specialist training programs in periodontology and implant dentistry. J. Clin. Periodontol..

[39] Colombo M., Mangano C., Mijiritsky E., Krebs M., Hauschild U., Fortin T. (2017). Clinical applications and effectiveness of guided implant surgery: A critical review based on randomized controlled trials. BMC Oral Health.

[40] Donos N., Akcali A., Padhye N., Sculean A., Calciolari E. (2023). Bone regeneration in implant dentistry: Which are the factors affecting the clinical outcome?. Periodontology 2000.

[41] Park Y.S., Lee B.A., Choi S.H., Kim Y.T. (2022). Evaluation of failed implants and reimplantation at sites of previous dental implant failure: Survival rates and risk factors. J. Periodontal. Implant. Sci..

[42] Fuglsig J.M.d.C.e.S., Reis I.N.R.d., Yeung A.W.K., Bornstein M.M., Spin-Neto R. (2024). The current role and future potential of digital diagnostic imaging in implant dentistry: A scoping review. Clin. Oral Implant. Res..

[43] Graziani F., Karapetsa D., Alonso B., Herrera D. (2017). Nonsurgical and surgical treatment of periodontitis: How many options for one disease?. Periodontology 2000.

[44] Mosaddad S.A., Talebi S., Keyhan S.O., Fallahi H.R., Darvishi M., Aghili S.S., Tavahodi N., Namanloo R.A., Heboyan A., Fathi A. (2024). Dental implant considerations in patients with systemic diseases: An updated comprehensive review. J. Oral Rehabil..

[45] Alsharif S.H., AlFada M., Alaqeel A.A. (2023). A retrospective review of Mohs micrographic surgery trends over more than 10 years in Saudi Arabia. Saudi Med. J..

[46] Linghu D., Zhang D., Liu M. (2025). Predictors of implant failure: A comprehensive analysis of risk factors in oral implant restoration for patients with partial defects of dentition. Biomol. Biomed..

